# [8-(Diphenyl­phosphan­yl)naphthyl-κ^2^ *C* ^1^,*P*](phenyl­ethyn­yl)tris­(trimethyl­phosphane-κ*P*)iron(II)

**DOI:** 10.1107/S1600536808041421

**Published:** 2008-12-13

**Authors:** Robert Beck, Hongjian Sun, Dexin Guan, Xiaoyan Li

**Affiliations:** a School of Chemistry and Chemical Engineering, Shandong University, Shanda Nanlu 27, 250100 Jinan, People’s Republic of China

## Abstract

The title compound, [Fe(C_8_H_5_)(C_22_H_16_P)(C_3_H_9_P)_3_], was synthesized by the addition of phenyl­ethine to a solution of the parent methyl iron complex Fe(CH_3_){P(C_6_H_5_)_2_(C_10_H_6_)}(PMe_3_)_3_ at 213 K, accompanied by evolution of methane. The coordination around the iron center can be described as slightly distorted octa­hedral [Fe—P 2.2485 (12)–2.2902 (12) Å; Fe—C 1.918 (5), 2.015 (4) Å], with a *meridional* arrangement of the trimethyl­phosphine ligands and the introduced terminal alkinyl-ligand *trans* to the P(Ph)_2_-anchoring group.

## Related literature

Some details of the synthesis of inter­mediates were described by Carré *et al.* (2000 ▶) and Karsch (1977 ▶). For related iron(II) complexes, see: Venturi *et al.* (2004 ▶); Costuas *et al.* (2004 ▶); Beck *et al.* (2008 ▶). Highly active iron(II) catalysts for olefin polymerization have bee prepared by Britovsek *et al.* (1998 ▶) and Small *et al.* (1998 ▶).
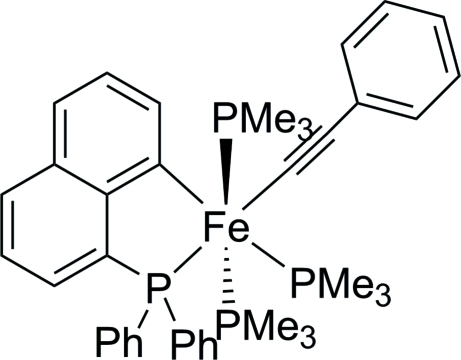


## Experimental

### 

#### Crystal data

[Fe(C_8_H_5_)(C_22_H_16_P)(C_3_H_9_P)_3_]
*M* *_r_* = 696.50
Monoclinic, 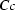

*a* = 9.6667 (18) Å
*b* = 19.965 (4) Å
*c* = 19.035 (4) Å
β = 99.322 (7)°
*V* = 3625.2 (12) Å^3^
*Z* = 4
Mo *K*α radiation
μ = 0.62 mm^−1^
*T* = 293 (2) K
0.15 × 0.13 × 0.10 mm

#### Data collection

Bruker SMART CCD area-detector diffractometer
Absorption correction: multi-scan (*SADABS*; Sheldrick, 2004 ▶) *T*_min_ = 0.913, *T*_max_ = 0.945
19984 measured reflections
5830 independent reflections
4723 reflections with *I* > 2σ(*I*)
*R*_int_ = 0.066

#### Refinement

*R*[*F* ^2^ > 2σ(*F* ^2^)] = 0.043
*wR*(*F* ^2^) = 0.098
*S* = 1.00
5830 reflections
406 parameters
2 restraints
H-atom parameters constrained
Δρ_max_ = 0.31 e Å^−3^
Δρ_min_ = −0.49 e Å^−3^
Absolute structure: Flack (1983 ▶); 2641 Friedel pairs
Flack parameter: 0.045 (17)

### 

Data collection: *SMART* (Bruker, 1997 ▶); cell refinement: *SAINT* (Bruker, 1997 ▶); data reduction: *SAINT*; program(s) used to solve structure: *SHELXS97* (Sheldrick, 2008 ▶); program(s) used to refine structure: *SHELXL97* (Sheldrick, 2008 ▶); molecular graphics: *DIAMOND* (Brandenburg & Berndt, 1999 ▶); software used to prepare material for publication: *SHELXTL* (Sheldrick, 2008 ▶).

## Supplementary Material

Crystal structure: contains datablocks I, global. DOI: 10.1107/S1600536808041421/cv2474sup1.cif

Structure factors: contains datablocks I. DOI: 10.1107/S1600536808041421/cv2474Isup2.hkl

Additional supplementary materials:  crystallographic information; 3D view; checkCIF report

## Figures and Tables

**Fig. 1.**
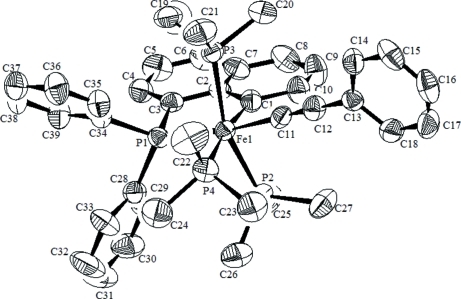
The molecular structure of (1) showing the atomic labels and 50% probability displacement ellipsoids. H atoms omitted for clarity.

## supplementary crystallographic information

### Comment

Recently, much attention has been paid to the use of highly active bis(imino) pyridine iron complexes for ethylene polymerization and alpha-olefine oligomerization as catalyst precursors reported by Britovsek *et al.* (1998) and Small *et al.* (1998). At this stage it is remarkable how little is known about this type of reactive intermediates. With labile methyl iron complexes in the presence P(Ph)_2_-anchoring groups we were able to synthesize five-membered metallacyles under smooth conditions (see Beck *et al.* 2008). These complexes represents model compounds for the catalytic functionalization of C,*C* bonds. Related, phenylethinyl-iron(II) complexes were prepared by Venturi *et al.* (2004) and Costuas *et al.* (2004). The title compound Fe(CCPh){P(C_6_H_5_)_2_(C_10_H_6_)}(PMe_3_)_3_ (1), was synthesized by addition of phenylethine to a solution of the parent methyl iron complex Fe(CH_3_){P(C_6_H_5_)_2_(C_10_H_6_)}(PMe_3_)_3_ at low temperature (213 K), accompanied by evolution of methane.

The *meridional-cis* arrangement of the ligands in the configuration remained stable when the reaction is finished, by means no rearrangement occurred because of different *trans* influence of the alkinyl carbon atom and no indication for isomers after the reaction was completed. From pentane solutions at 253 K orange-red crystal in form of needles were obtained, which were suitable for X-ray diffraction. The molecular structure of 1 is shown in Figure 1. The iron atom attains an octahedral coordination with a *meridional* arrangement of the trimethylphosphine ligands by two *trans* PMe_3_ orientated groups (P3—Fe1—P2 = 158.97 (5)°) a *cis* disposed PMe_3_ group (P4) *trans* to the metallated carbon (C1) atom (C1—Fe1—P4 = 174.38 (14)°), and the chelating phosphorus atom (P1) *trans* to the alkinyl group (CCPh) with an angle of (C11—Fe1—P1 = 173.69 (14)°). The inner bond angles of the trimethylphosphine ligands are close to 90°, with the smallest involving the bite angle of the five-membered chelate ring (C1—Fe1—P1 = 84.07 (13)°). The sum of internal angles is 537°, indicating considerable relaxation of the metallacycle towards planarity. The Fe—P bond lengths fall within the range observed for other Fe(II) complexes containing PMe_3_ groups (Venturi *et al.*, 2004). The complex contains two different Fe—C bonds with *sp*, and *sp*^2^ hybridization with bond lengths of 1.918 (5) and 2.015 (4) Å, respectively, which are consistent with those in related Cp-stabilized iron(II) complexes with terminal alkinyl groups reported by Costuas *et al.* (2004).

### Experimental

Standard vacuum techniques were used in manipulations of volatile and air sensitive material. Literature methods were applied in the preparation of dimethyltetrakis(trimethylphosphine)iron(II) (Karsch, 1977), and 1-Diphenylphosphanyl-naphthaline (Carré *et al.*, 2000). Other chemicals were used as purchased. The title compound was synthesized by combining a solution of phenylethine (140 mg, 1.37 mmol) in 50 ml of THF at -70 °C with a sample of Fe(CH_3_){P(C_6_H_5_)_2_(C_10_H_6_)}(PMe_3_)_3_ (836 mg, 1.37 mmol) in 50 ml of THF, effecting a change of color from red to orange. After warm-up the mixture was kept stirring at 293 K for 16 h, and then the volatiles were removed *in vacuo* to give orange solid. This was dissolved in 50 ml of pentane and crystallized at 253 K to give yellow crystals, which were suitable for X-ray diffraction. Yield 391 mg (41%); 396–398 K (dec.).

### Refinement

All H atoms were fixed geometrically and treated as riding on their parent atoms with C—H = 0.93 Å (aromatic) and 0.96 Å (methyl), and with *U*_iso_(H) = 1.2 (1.5 for methyl groups) times *U*_eq_(C).

### Crystal data

**Table:**

[Fe(C_8_H_5_)(C_22_H_16_P)(C_3_H_9_P)_3_]	*F*(000) = 1472
*M**_r_* = 696.50	*D*_x_ = 1.276 Mg m^−^^3^
Monoclinic, *C**c*	Melting point: 398 K
Hall symbol: C -2yc	Mo *K*α radiation, λ = 0.71073 Å
*a* = 9.6667 (18) Å	Cell parameters from 1391 reflections
*b* = 19.965 (4) Å	θ = 1.9–27.3°
*c* = 19.035 (4) Å	µ = 0.62 mm^−^^1^
β = 99.322 (7)°	*T* = 293 K
*V* = 3625.2 (12) Å^3^	Block, orange
*Z* = 4	0.15 × 0.13 × 0.10 mm

### Data collection

**Table:**

Bruker SMART CCD area-detector diffractometer	5830 independent reflections
Radiation source: fine-focus sealed tube	4723 reflections with *I* > 2σ(*I*)
graphite	*R*_int_ = 0.066
phi and ω scans	θ_max_ = 25.0°, θ_min_ = 2.0°
Absorption correction: multi-scan (*SADABS*; Sheldrick, 2004)	*h* = −11→11
*T*_min_ = 0.913, *T*_max_ = 0.945	*k* = −21→23
19984 measured reflections	*l* = −22→22

### Refinement

**Table:**

Refinement on *F*^2^	Secondary atom site location: difference Fourier map
Least-squares matrix: full	Hydrogen site location: inferred from neighbouring sites
*R*[*F*^2^ > 2σ(*F*^2^)] = 0.043	H-atom parameters constrained
*wR*(*F*^2^) = 0.098	*w* = 1/[σ^2^(*F*_o_^2^) + (0.047*P*)^2^ + 0.0194*P*] where *P* = (*F*_o_^2^ + 2*F*_c_^2^)/3
*S* = 1.00	(Δ/σ)_max_ = 0.003
5830 reflections	Δρ_max_ = 0.31 e Å^−^^3^
406 parameters	Δρ_min_ = −0.49 e Å^−^^3^
2 restraints	Absolute structure: Flack (1983); 2641 Friedel pairs
Primary atom site location: structure-invariant direct methods	Flack parameter: 0.045 (17)

### Special details

**Table:**

Geometry. All e.s.d.'s (except the e.s.d. in the dihedral angle between two l.s. planes) are estimated using the full covariance matrix. The cell e.s.d.'s are taken into account individually in the estimation of e.s.d.'s in distances, angles and torsion angles; correlations between e.s.d.'s in cell parameters are only used when they are defined by crystal symmetry. An approximate (isotropic) treatment of cell e.s.d.'s is used for estimating e.s.d.'s involving l.s. planes.
Refinement. Refinement of *F*^2^ against ALL reflections. The weighted *R*-factor *wR* and goodness of fit *S* are based on *F*^2^, conventional *R*-factors *R* are based on *F*, with *F* set to zero for negative *F*^2^. The threshold expression of *F*^2^ > σ(*F*^2^) is used only for calculating *R*-factors(gt) *etc*. and is not relevant to the choice of reflections for refinement. *R*-factors based on *F*^2^ are statistically about twice as large as those based on *F*, and *R*- factors based on ALL data will be even larger.

### Fractional atomic coordinates and isotropic or equivalent isotropic displacement parameters (Å^2^)

**Table:**

	*x*	*y*	*z*	*U*_iso_*/*U*_eq_	
Fe1	0.57710 (4)	0.97187 (3)	−0.00279 (3)	0.03402 (15)	
P1	0.73164 (11)	0.89846 (6)	0.05495 (5)	0.0365 (3)	
P2	0.42146 (11)	0.96230 (6)	0.07337 (6)	0.0445 (3)	
P3	0.73799 (11)	1.02026 (6)	−0.06302 (6)	0.0415 (3)	
P4	0.44623 (13)	0.90402 (7)	−0.08487 (6)	0.0484 (3)	
C1	0.6666 (5)	1.0366 (2)	0.0723 (2)	0.0393 (10)	
C2	0.7904 (4)	1.0176 (2)	0.12015 (19)	0.0369 (10)	
C3	0.8470 (4)	0.9530 (2)	0.1165 (2)	0.0385 (10)	
C4	0.9682 (5)	0.9347 (2)	0.1606 (2)	0.0473 (11)	
H4	1.0048	0.8919	0.1571	0.057*	
C5	1.0364 (5)	0.9798 (3)	0.2105 (2)	0.0575 (14)	
H5	1.1195	0.9673	0.2392	0.069*	
C6	0.9829 (5)	1.0414 (3)	0.2174 (2)	0.0549 (13)	
H6	1.0288	1.0704	0.2516	0.066*	
C7	0.8580 (5)	1.0626 (2)	0.1735 (2)	0.0453 (11)	
C8	0.7988 (6)	1.1257 (3)	0.1804 (2)	0.0577 (14)	
H8	0.8412	1.1553	0.2150	0.069*	
C9	0.6789 (6)	1.1436 (3)	0.1365 (3)	0.0610 (14)	
H9	0.6382	1.1851	0.1423	0.073*	
C10	0.6157 (5)	1.0999 (2)	0.0822 (2)	0.0498 (12)	
H10	0.5363	1.1144	0.0518	0.060*	
C11	0.4624 (4)	1.0389 (2)	−0.0559 (2)	0.0404 (11)	
C12	0.3897 (5)	1.0786 (2)	−0.0953 (2)	0.0458 (11)	
C13	0.3001 (4)	1.1223 (2)	−0.1402 (2)	0.0386 (10)	
C14	0.3382 (5)	1.1498 (2)	−0.2021 (2)	0.0504 (12)	
H14	0.4253	1.1396	−0.2141	0.060*	
C15	0.2487 (7)	1.1918 (2)	−0.2458 (2)	0.0632 (15)	
H15	0.2769	1.2102	−0.2860	0.076*	
C16	0.1194 (7)	1.2065 (3)	−0.2303 (3)	0.0667 (15)	
H16	0.0590	1.2343	−0.2602	0.080*	
C17	0.0789 (5)	1.1804 (3)	−0.1706 (3)	0.0642 (14)	
H17	−0.0097	1.1900	−0.1602	0.077*	
C18	0.1684 (5)	1.1397 (2)	−0.1254 (3)	0.0533 (12)	
H18	0.1401	1.1235	−0.0842	0.064*	
C19	0.9258 (5)	1.0057 (3)	−0.0370 (3)	0.0612 (14)	
H19A	0.9456	0.9589	−0.0418	0.092*	
H19B	0.9542	1.0191	0.0116	0.092*	
H19C	0.9765	1.0314	−0.0671	0.092*	
C20	0.7453 (6)	1.1119 (2)	−0.0592 (3)	0.0710 (15)	
H20A	0.7928	1.1284	−0.0963	0.107*	
H20B	0.7953	1.1258	−0.0138	0.107*	
H20C	0.6518	1.1297	−0.0656	0.107*	
C21	0.7253 (6)	1.0122 (3)	−0.1594 (2)	0.0716 (16)	
H21A	0.7990	1.0375	−0.1751	0.107*	
H21B	0.6362	1.0289	−0.1823	0.107*	
H21C	0.7340	0.9659	−0.1716	0.107*	
C22	0.4704 (6)	0.9031 (3)	−0.1791 (2)	0.0835 (19)	
H22A	0.5626	0.8868	−0.1824	0.125*	
H22B	0.4596	0.9476	−0.1981	0.125*	
H22C	0.4016	0.8742	−0.2057	0.125*	
C23	0.2569 (6)	0.9208 (3)	−0.1019 (3)	0.0697 (16)	
H23A	0.2120	0.8903	−0.1374	0.105*	
H23B	0.2408	0.9659	−0.1184	0.105*	
H23C	0.2191	0.9148	−0.0587	0.105*	
C24	0.4422 (6)	0.8136 (3)	−0.0715 (3)	0.0725 (17)	
H24A	0.4217	0.8043	−0.0247	0.109*	
H24B	0.5317	0.7948	−0.0761	0.109*	
H24C	0.3710	0.7941	−0.1065	0.109*	
C25	0.4800 (6)	0.9780 (3)	0.1683 (2)	0.0638 (15)	
H25A	0.5080	1.0239	0.1753	0.096*	
H25B	0.5580	0.9494	0.1855	0.096*	
H25C	0.4046	0.9688	0.1941	0.096*	
C26	0.3291 (6)	0.8838 (3)	0.0838 (3)	0.0706 (16)	
H26A	0.2769	0.8880	0.1224	0.106*	
H26B	0.3959	0.8481	0.0938	0.106*	
H26C	0.2660	0.8741	0.0407	0.106*	
C27	0.2763 (5)	1.0220 (3)	0.0600 (3)	0.0677 (16)	
H27A	0.2163	1.0141	0.0947	0.101*	
H27B	0.2238	1.0165	0.0131	0.101*	
H27C	0.3127	1.0668	0.0653	0.101*	
C28	0.6955 (5)	0.8309 (2)	0.1165 (2)	0.0443 (11)	
C29	0.7102 (5)	0.8412 (3)	0.1906 (2)	0.0558 (13)	
H29	0.7395	0.8827	0.2097	0.067*	
C30	0.6813 (6)	0.7903 (3)	0.2350 (3)	0.0701 (16)	
H30	0.6910	0.7977	0.2838	0.084*	
C31	0.6384 (7)	0.7290 (4)	0.2078 (4)	0.088 (2)	
H31	0.6192	0.6951	0.2383	0.106*	
C32	0.6232 (7)	0.7169 (3)	0.1358 (4)	0.091 (2)	
H32	0.5930	0.6752	0.1175	0.109*	
C33	0.6539 (6)	0.7681 (3)	0.0904 (3)	0.0659 (15)	
H33	0.6462	0.7597	0.0418	0.079*	
C34	0.8435 (4)	0.8478 (2)	0.0054 (2)	0.0404 (10)	
C35	0.8159 (5)	0.8479 (2)	−0.0685 (2)	0.0502 (12)	
H35	0.7431	0.8740	−0.0920	0.060*	
C36	0.8947 (6)	0.8099 (3)	−0.1076 (3)	0.0643 (15)	
H36	0.8734	0.8103	−0.1571	0.077*	
C37	1.0031 (6)	0.7718 (3)	−0.0754 (3)	0.0642 (14)	
H37	1.0567	0.7469	−0.1023	0.077*	
C38	1.0324 (5)	0.7704 (2)	−0.0022 (3)	0.0616 (14)	
H38	1.1056	0.7441	0.0204	0.074*	
C39	0.9539 (5)	0.8080 (2)	0.0377 (2)	0.0554 (13)	
H39	0.9752	0.8067	0.0871	0.066*	

### Atomic displacement parameters (Å^2^)

**Table:**

	*U*^11^	*U*^22^	*U*^33^	*U*^12^	*U*^13^	*U*^23^
Fe1	0.0313 (3)	0.0370 (3)	0.0331 (3)	0.0010 (3)	0.0032 (2)	0.0010 (3)
P1	0.0365 (6)	0.0344 (7)	0.0374 (5)	0.0002 (5)	0.0027 (4)	0.0021 (5)
P2	0.0356 (6)	0.0585 (8)	0.0404 (6)	0.0014 (6)	0.0090 (5)	0.0033 (6)
P3	0.0362 (6)	0.0459 (7)	0.0431 (6)	0.0010 (5)	0.0087 (5)	0.0066 (5)
P4	0.0468 (7)	0.0534 (8)	0.0419 (6)	−0.0074 (6)	−0.0024 (5)	−0.0050 (6)
C1	0.045 (3)	0.039 (3)	0.036 (2)	0.004 (2)	0.0099 (18)	0.0032 (19)
C2	0.039 (2)	0.037 (3)	0.036 (2)	−0.006 (2)	0.0091 (17)	0.0010 (18)
C3	0.038 (2)	0.042 (3)	0.036 (2)	−0.003 (2)	0.0074 (18)	0.0065 (19)
C4	0.041 (3)	0.047 (3)	0.051 (2)	0.001 (2)	−0.001 (2)	0.009 (2)
C5	0.052 (3)	0.068 (4)	0.046 (3)	−0.009 (3)	−0.013 (2)	0.007 (2)
C6	0.052 (3)	0.059 (4)	0.048 (3)	−0.014 (3)	−0.008 (2)	−0.007 (2)
C7	0.051 (3)	0.041 (3)	0.042 (2)	−0.012 (2)	0.0012 (19)	−0.002 (2)
C8	0.075 (4)	0.046 (3)	0.048 (3)	−0.014 (3)	−0.004 (2)	−0.011 (2)
C9	0.075 (4)	0.044 (3)	0.062 (3)	0.009 (3)	0.005 (3)	−0.016 (2)
C10	0.050 (3)	0.048 (3)	0.049 (2)	0.006 (2)	−0.001 (2)	−0.004 (2)
C11	0.033 (2)	0.045 (3)	0.044 (2)	0.001 (2)	0.0092 (19)	−0.009 (2)
C12	0.040 (3)	0.050 (3)	0.045 (2)	0.003 (2)	−0.001 (2)	−0.001 (2)
C13	0.037 (2)	0.035 (3)	0.042 (2)	−0.004 (2)	0.0024 (18)	−0.0018 (19)
C14	0.058 (3)	0.043 (3)	0.052 (3)	0.010 (2)	0.013 (2)	−0.001 (2)
C15	0.104 (5)	0.039 (3)	0.044 (2)	0.004 (3)	0.007 (3)	0.004 (2)
C16	0.077 (4)	0.047 (3)	0.069 (3)	0.020 (3)	−0.009 (3)	0.002 (3)
C17	0.043 (3)	0.061 (4)	0.086 (4)	0.016 (3)	0.002 (3)	0.000 (3)
C18	0.045 (3)	0.054 (3)	0.061 (3)	0.002 (2)	0.008 (2)	0.009 (2)
C19	0.043 (3)	0.067 (4)	0.075 (3)	−0.005 (3)	0.015 (2)	0.021 (3)
C20	0.068 (4)	0.055 (3)	0.095 (4)	−0.006 (3)	0.030 (3)	0.016 (3)
C21	0.069 (4)	0.097 (5)	0.052 (3)	−0.008 (3)	0.019 (2)	0.014 (3)
C22	0.080 (4)	0.125 (6)	0.041 (3)	−0.011 (4)	0.000 (3)	−0.011 (3)
C23	0.055 (3)	0.079 (4)	0.068 (3)	−0.003 (3)	−0.010 (3)	−0.004 (3)
C24	0.069 (4)	0.065 (4)	0.079 (4)	−0.014 (3)	0.000 (3)	−0.019 (3)
C25	0.060 (3)	0.087 (4)	0.046 (3)	0.009 (3)	0.016 (2)	0.001 (3)
C26	0.065 (4)	0.083 (4)	0.067 (3)	−0.009 (3)	0.023 (3)	0.008 (3)
C27	0.045 (3)	0.096 (5)	0.064 (3)	0.015 (3)	0.015 (2)	0.010 (3)
C28	0.038 (2)	0.046 (3)	0.048 (2)	0.001 (2)	0.0051 (18)	0.009 (2)
C29	0.051 (3)	0.065 (3)	0.051 (3)	−0.007 (3)	0.007 (2)	0.016 (2)
C30	0.070 (4)	0.085 (5)	0.057 (3)	−0.010 (3)	0.017 (3)	0.021 (3)
C31	0.089 (5)	0.085 (5)	0.091 (5)	−0.019 (4)	0.014 (4)	0.043 (4)
C32	0.097 (5)	0.053 (4)	0.119 (6)	−0.026 (3)	0.007 (4)	0.022 (4)
C33	0.080 (4)	0.042 (3)	0.073 (3)	−0.012 (3)	0.006 (3)	0.006 (3)
C34	0.042 (2)	0.037 (3)	0.043 (2)	0.002 (2)	0.0089 (19)	−0.001 (2)
C35	0.054 (3)	0.047 (3)	0.049 (2)	0.008 (2)	0.005 (2)	0.000 (2)
C36	0.086 (4)	0.057 (3)	0.050 (3)	0.012 (3)	0.013 (3)	−0.014 (2)
C37	0.073 (4)	0.050 (3)	0.075 (4)	0.006 (3)	0.029 (3)	−0.013 (3)
C38	0.062 (4)	0.047 (3)	0.077 (3)	0.020 (2)	0.013 (3)	0.003 (3)
C39	0.062 (3)	0.053 (3)	0.052 (3)	0.015 (3)	0.009 (2)	−0.001 (2)

### Geometric parameters (Å, °)

**Table:**

Fe1—C11	1.918 (5)	C19—H19B	0.9600
Fe1—C1	2.015 (4)	C19—H19C	0.9600
Fe1—P1	2.2485 (12)	C20—H20A	0.9600
Fe1—P2	2.2601 (12)	C20—H20B	0.9600
Fe1—P4	2.2882 (13)	C20—H20C	0.9600
Fe1—P3	2.2902 (12)	C21—H21A	0.9600
P1—C3	1.838 (4)	C21—H21B	0.9600
P1—C34	1.846 (4)	C21—H21C	0.9600
P1—C28	1.857 (4)	C22—H22A	0.9600
P2—C27	1.827 (5)	C22—H22B	0.9600
P2—C26	1.831 (6)	C22—H22C	0.9600
P2—C25	1.832 (5)	C23—H23A	0.9600
P3—C19	1.827 (5)	C23—H23B	0.9600
P3—C21	1.826 (5)	C23—H23C	0.9600
P3—C20	1.833 (5)	C24—H24A	0.9600
P4—C24	1.825 (6)	C24—H24B	0.9600
P4—C23	1.836 (6)	C24—H24C	0.9600
P4—C22	1.846 (5)	C25—H25A	0.9600
C1—C10	1.380 (6)	C25—H25B	0.9600
C1—C2	1.434 (6)	C25—H25C	0.9600
C2—C3	1.407 (6)	C26—H26A	0.9600
C2—C7	1.432 (6)	C26—H26B	0.9600
C3—C4	1.375 (6)	C26—H26C	0.9600
C4—C5	1.395 (6)	C27—H27A	0.9600
C4—H4	0.9300	C27—H27B	0.9600
C5—C6	1.348 (7)	C27—H27C	0.9600
C5—H5	0.9300	C28—C33	1.384 (6)
C6—C7	1.417 (6)	C28—C29	1.410 (6)
C6—H6	0.9300	C29—C30	1.380 (7)
C7—C8	1.398 (7)	C29—H29	0.9300
C8—C9	1.363 (7)	C30—C31	1.367 (8)
C8—H8	0.9300	C30—H30	0.9300
C9—C10	1.413 (6)	C31—C32	1.376 (9)
C9—H9	0.9300	C31—H31	0.9300
C10—H10	0.9300	C32—C33	1.401 (8)
C11—C12	1.230 (6)	C32—H32	0.9300
C12—C13	1.416 (6)	C33—H33	0.9300
C13—C18	1.392 (6)	C34—C35	1.390 (6)
C13—C14	1.402 (6)	C34—C39	1.392 (6)
C14—C15	1.381 (7)	C35—C36	1.376 (7)
C14—H14	0.9300	C35—H35	0.9300
C15—C16	1.362 (8)	C36—C37	1.358 (7)
C15—H15	0.9300	C36—H36	0.9300
C16—C17	1.364 (8)	C37—C38	1.375 (7)
C16—H16	0.9300	C37—H37	0.9300
C17—C18	1.380 (6)	C38—C39	1.379 (7)
C17—H17	0.9300	C38—H38	0.9300
C18—H18	0.9300	C39—H39	0.9300
C19—H19A	0.9600		
			
C11—Fe1—C1	94.06 (16)	P3—C19—H19B	109.5
C11—Fe1—P1	173.69 (14)	H19A—C19—H19B	109.5
C1—Fe1—P1	84.07 (13)	P3—C19—H19C	109.5
C11—Fe1—P2	90.51 (13)	H19A—C19—H19C	109.5
C1—Fe1—P2	81.56 (13)	H19B—C19—H19C	109.5
P1—Fe1—P2	95.16 (5)	P3—C20—H20A	109.5
C11—Fe1—P4	81.08 (13)	P3—C20—H20B	109.5
C1—Fe1—P4	171.99 (14)	H20A—C20—H20B	109.5
P1—Fe1—P4	101.42 (5)	P3—C20—H20C	109.5
P2—Fe1—P4	92.07 (5)	H20A—C20—H20C	109.5
C11—Fe1—P3	79.82 (13)	H20B—C20—H20C	109.5
C1—Fe1—P3	80.56 (13)	P3—C21—H21A	109.5
P1—Fe1—P3	93.92 (5)	P3—C21—H21B	109.5
P2—Fe1—P3	158.97 (5)	H21A—C21—H21B	109.5
P4—Fe1—P3	104.66 (5)	P3—C21—H21C	109.5
C3—P1—C34	107.7 (2)	H21A—C21—H21C	109.5
C3—P1—C28	100.65 (19)	H21B—C21—H21C	109.5
C34—P1—C28	96.3 (2)	P4—C22—H22A	109.5
C3—P1—Fe1	102.26 (14)	P4—C22—H22B	109.5
C34—P1—Fe1	120.34 (13)	H22A—C22—H22B	109.5
C28—P1—Fe1	127.17 (15)	P4—C22—H22C	109.5
C27—P2—C26	101.3 (3)	H22A—C22—H22C	109.5
C27—P2—C25	97.7 (2)	H22B—C22—H22C	109.5
C26—P2—C25	96.7 (3)	P4—C23—H23A	109.5
C27—P2—Fe1	115.56 (18)	P4—C23—H23B	109.5
C26—P2—Fe1	121.94 (19)	H23A—C23—H23B	109.5
C25—P2—Fe1	119.18 (18)	P4—C23—H23C	109.5
C19—P3—C21	99.2 (3)	H23A—C23—H23C	109.5
C19—P3—C20	96.8 (3)	H23B—C23—H23C	109.5
C21—P3—C20	97.1 (3)	P4—C24—H24A	109.5
C19—P3—Fe1	121.53 (17)	P4—C24—H24B	109.5
C21—P3—Fe1	121.69 (19)	H24A—C24—H24B	109.5
C20—P3—Fe1	115.26 (19)	P4—C24—H24C	109.5
C24—P4—C23	99.3 (3)	H24A—C24—H24C	109.5
C24—P4—C22	97.7 (3)	H24B—C24—H24C	109.5
C23—P4—C22	96.3 (3)	P2—C25—H25A	109.5
C24—P4—Fe1	120.82 (17)	P2—C25—H25B	109.5
C23—P4—Fe1	115.95 (19)	H25A—C25—H25B	109.5
C22—P4—Fe1	121.8 (2)	P2—C25—H25C	109.5
C10—C1—C2	115.8 (4)	H25A—C25—H25C	109.5
C10—C1—Fe1	124.3 (3)	H25B—C25—H25C	109.5
C2—C1—Fe1	119.9 (3)	P2—C26—H26A	109.5
C3—C2—C7	118.3 (4)	P2—C26—H26B	109.5
C3—C2—C1	120.2 (4)	H26A—C26—H26B	109.5
C7—C2—C1	121.5 (4)	P2—C26—H26C	109.5
C4—C3—C2	120.9 (4)	H26A—C26—H26C	109.5
C4—C3—P1	126.9 (4)	H26B—C26—H26C	109.5
C2—C3—P1	111.9 (3)	P2—C27—H27A	109.5
C3—C4—C5	120.4 (4)	P2—C27—H27B	109.5
C3—C4—H4	119.8	H27A—C27—H27B	109.5
C5—C4—H4	119.8	P2—C27—H27C	109.5
C6—C5—C4	120.6 (4)	H27A—C27—H27C	109.5
C6—C5—H5	119.7	H27B—C27—H27C	109.5
C4—C5—H5	119.7	C33—C28—C29	117.9 (4)
C5—C6—C7	121.3 (4)	C33—C28—P1	120.4 (3)
C5—C6—H6	119.4	C29—C28—P1	121.7 (4)
C7—C6—H6	119.4	C30—C29—C28	120.5 (5)
C8—C7—C6	122.5 (4)	C30—C29—H29	119.7
C8—C7—C2	119.1 (4)	C28—C29—H29	119.7
C6—C7—C2	118.5 (4)	C31—C30—C29	120.4 (5)
C9—C8—C7	120.0 (4)	C31—C30—H30	119.8
C9—C8—H8	120.0	C29—C30—H30	119.8
C7—C8—H8	120.0	C30—C31—C32	120.8 (5)
C8—C9—C10	120.6 (5)	C30—C31—H31	119.6
C8—C9—H9	119.7	C32—C31—H31	119.6
C10—C9—H9	119.7	C31—C32—C33	119.1 (6)
C1—C10—C9	123.0 (4)	C31—C32—H32	120.4
C1—C10—H10	118.5	C33—C32—H32	120.4
C9—C10—H10	118.5	C28—C33—C32	121.2 (5)
C12—C11—Fe1	174.3 (4)	C28—C33—H33	119.4
C11—C12—C13	177.2 (5)	C32—C33—H33	119.4
C18—C13—C14	116.5 (4)	C35—C34—C39	117.1 (4)
C18—C13—C12	121.4 (4)	C35—C34—P1	119.0 (3)
C14—C13—C12	122.1 (4)	C39—C34—P1	123.9 (3)
C15—C14—C13	121.3 (5)	C36—C35—C34	121.0 (4)
C15—C14—H14	119.4	C36—C35—H35	119.5
C13—C14—H14	119.4	C34—C35—H35	119.5
C16—C15—C14	120.5 (5)	C37—C36—C35	121.2 (5)
C16—C15—H15	119.7	C37—C36—H36	119.4
C14—C15—H15	119.7	C35—C36—H36	119.4
C15—C16—C17	119.7 (5)	C36—C37—C38	119.1 (5)
C15—C16—H16	120.2	C36—C37—H37	120.5
C17—C16—H16	120.2	C38—C37—H37	120.5
C16—C17—C18	120.5 (5)	C37—C38—C39	120.4 (5)
C16—C17—H17	119.7	C37—C38—H38	119.8
C18—C17—H17	119.7	C39—C38—H38	119.8
C17—C18—C13	121.5 (5)	C38—C39—C34	121.2 (4)
C17—C18—H18	119.3	C38—C39—H39	119.4
C13—C18—H18	119.3	C34—C39—H39	119.4
P3—C19—H19A	109.5		
			
C1—Fe1—P1—C3	−10.24 (18)	C10—C1—C2—C7	−1.6 (6)
P2—Fe1—P1—C3	−91.18 (14)	Fe1—C1—C2—C7	179.1 (3)
P4—Fe1—P1—C3	175.66 (14)	C7—C2—C3—C4	−3.3 (6)
P3—Fe1—P1—C3	69.83 (14)	C1—C2—C3—C4	177.9 (4)
C1—Fe1—P1—C34	−129.5 (2)	C7—C2—C3—P1	171.0 (3)
P2—Fe1—P1—C34	149.58 (16)	C1—C2—C3—P1	−7.8 (5)
P4—Fe1—P1—C34	56.41 (17)	C34—P1—C3—C4	−46.0 (4)
P3—Fe1—P1—C34	−49.41 (16)	C28—P1—C3—C4	54.1 (4)
C1—Fe1—P1—C28	103.5 (2)	Fe1—P1—C3—C4	−173.8 (4)
P2—Fe1—P1—C28	22.53 (19)	C34—P1—C3—C2	140.1 (3)
P4—Fe1—P1—C28	−70.63 (19)	C28—P1—C3—C2	−119.7 (3)
P3—Fe1—P1—C28	−176.46 (19)	Fe1—P1—C3—C2	12.3 (3)
C11—Fe1—P2—C27	−5.6 (3)	C2—C3—C4—C5	0.8 (7)
C1—Fe1—P2—C27	88.4 (2)	P1—C3—C4—C5	−172.6 (4)
P1—Fe1—P2—C27	171.6 (2)	C3—C4—C5—C6	1.6 (7)
P4—Fe1—P2—C27	−86.7 (2)	C4—C5—C6—C7	−1.3 (8)
P3—Fe1—P2—C27	56.4 (3)	C5—C6—C7—C8	178.7 (5)
C11—Fe1—P2—C26	118.1 (3)	C5—C6—C7—C2	−1.2 (7)
C1—Fe1—P2—C26	−147.9 (2)	C3—C2—C7—C8	−176.5 (4)
P1—Fe1—P2—C26	−64.7 (2)	C1—C2—C7—C8	2.3 (6)
P4—Fe1—P2—C26	37.0 (2)	C3—C2—C7—C6	3.5 (6)
P3—Fe1—P2—C26	−179.9 (2)	C1—C2—C7—C6	−177.8 (4)
C11—Fe1—P2—C25	−121.5 (2)	C6—C7—C8—C9	179.6 (5)
C1—Fe1—P2—C25	−27.5 (2)	C2—C7—C8—C9	−0.4 (7)
P1—Fe1—P2—C25	55.7 (2)	C7—C8—C9—C10	−2.0 (8)
P4—Fe1—P2—C25	157.4 (2)	C2—C1—C10—C9	−0.9 (7)
P3—Fe1—P2—C25	−59.5 (2)	Fe1—C1—C10—C9	178.4 (4)
C11—Fe1—P3—C19	165.2 (3)	C8—C9—C10—C1	2.8 (8)
C1—Fe1—P3—C19	69.2 (3)	C18—C13—C14—C15	−0.1 (7)
P1—Fe1—P3—C19	−14.1 (2)	C12—C13—C14—C15	179.3 (4)
P2—Fe1—P3—C19	101.4 (3)	C13—C14—C15—C16	−1.3 (7)
P4—Fe1—P3—C19	−117.0 (2)	C14—C15—C16—C17	1.0 (8)
C11—Fe1—P3—C21	−68.0 (3)	C15—C16—C17—C18	0.7 (8)
C1—Fe1—P3—C21	−163.9 (3)	C16—C17—C18—C13	−2.1 (8)
P1—Fe1—P3—C21	112.8 (2)	C14—C13—C18—C17	1.8 (7)
P2—Fe1—P3—C21	−131.8 (3)	C12—C13—C18—C17	−177.6 (4)
P4—Fe1—P3—C21	9.9 (2)	C3—P1—C28—C33	−157.4 (4)
C11—Fe1—P3—C20	48.9 (3)	C34—P1—C28—C33	−48.0 (4)
C1—Fe1—P3—C20	−47.0 (3)	Fe1—P1—C28—C33	88.2 (4)
P1—Fe1—P3—C20	−130.3 (2)	C3—P1—C28—C29	22.2 (4)
P2—Fe1—P3—C20	−14.9 (3)	C34—P1—C28—C29	131.6 (4)
P4—Fe1—P3—C20	126.8 (2)	Fe1—P1—C28—C29	−92.3 (4)
C11—Fe1—P4—C24	−165.1 (3)	C33—C28—C29—C30	−1.1 (7)
P1—Fe1—P4—C24	20.8 (2)	P1—C28—C29—C30	179.3 (4)
P2—Fe1—P4—C24	−74.9 (2)	C28—C29—C30—C31	0.2 (9)
P3—Fe1—P4—C24	117.9 (2)	C29—C30—C31—C32	0.0 (10)
C11—Fe1—P4—C23	−45.2 (2)	C30—C31—C32—C33	0.7 (11)
P1—Fe1—P4—C23	140.7 (2)	C29—C28—C33—C32	1.8 (8)
P2—Fe1—P4—C23	45.0 (2)	P1—C28—C33—C32	−178.6 (5)
P3—Fe1—P4—C23	−122.1 (2)	C31—C32—C33—C28	−1.7 (10)
C11—Fe1—P4—C22	71.3 (3)	C3—P1—C34—C35	−126.4 (4)
P1—Fe1—P4—C22	−102.8 (3)	C28—P1—C34—C35	130.3 (4)
P2—Fe1—P4—C22	161.5 (3)	Fe1—P1—C34—C35	−10.0 (4)
P3—Fe1—P4—C22	−5.7 (3)	C3—P1—C34—C39	54.7 (4)
C11—Fe1—C1—C10	15.3 (4)	C28—P1—C34—C39	−48.6 (4)
P1—Fe1—C1—C10	−170.7 (4)	Fe1—P1—C34—C39	171.2 (3)
P2—Fe1—C1—C10	−74.6 (4)	C39—C34—C35—C36	0.2 (7)
P3—Fe1—C1—C10	94.3 (4)	P1—C34—C35—C36	−178.7 (4)
C11—Fe1—C1—C2	−165.4 (3)	C34—C35—C36—C37	−0.9 (8)
P1—Fe1—C1—C2	8.5 (3)	C35—C36—C37—C38	1.2 (8)
P2—Fe1—C1—C2	104.7 (3)	C36—C37—C38—C39	−0.8 (8)
P3—Fe1—C1—C2	−86.5 (3)	C37—C38—C39—C34	0.2 (8)
C10—C1—C2—C3	177.1 (4)	C35—C34—C39—C38	0.1 (7)
Fe1—C1—C2—C3	−2.2 (5)	P1—C34—C39—C38	179.0 (4)
